# Imaging features and differences among the three primary malignant non-Wilms tumors in children

**DOI:** 10.1186/s12880-021-00715-z

**Published:** 2021-12-01

**Authors:** Yupeng Zhu, Wangxing Fu, Yangyue Huang, Ning Sun, Yun Peng

**Affiliations:** 1 Department of Radiology, Beijing Children’s Hospital, Capital Medical University, National Center for Children’s Health, 56 Nanlishi Road, Xicheng District, Beijing, China 100045; 2 Department of Pediatric Urology, Beijing Children’s Hospital, Capital Medical University, National Center for Children’s Health, 56 Nanlishi Road, Xicheng District, Beijing, China 100045

**Keywords:** Children, Renal tumor, Clear cell sarcoma of kidney, Malignant rhabdomyoma tumor of kidney, Renal cell carcinoma, Imaging

## Abstract

**Background:**

The pathology, treatment and prognosis of malignant non-Wilms tumors (NWTs) are different, so it is necessary to differentiate these types of tumors. The purpose of this study was to review the clinical and imaging features of malignant NWTs and features of tumor metastasis.

**Methods:**

We retrospectively analyzed the CT images of 65 pediatric patients with NWTs from March 2008 to July 2020, mainly including clear cell sarcoma of the kidney (CCSK), malignant rhabdomyoma tumor of the kidney (MRTK) and renal cell carcinoma (RCC). Available pretreatment contrast-enhanced abdominal CT examinations were reviewed. The clinical features of the patients, imaging findings of the primary mass, and locoregional metastasis patterns were evaluated in correlation with pathological and surgical findings.

**Results:**

The study included CCSK (22 cases), MRTK (27 cases) and RCC (16 cases). There were no significant differences observed among the sex ratios of CCSK, MRTK and RCC (all *P* > 0.05). Among the three tumors, the onset age of MRTK patients was the smallest, while that of RCC patients was the largest (all *P* < 0.05). The tumor diameter of CCSK was larger than that of MRTK and RCC (all *P* < 0.001). For hemorrhage and necrosis, the proportion of MRTK patients was larger than that of the other two tumors (*P* = 0.017). For calcification in tumors, the proportion of calcification in RCC was highest (*P* = 0.009). Only MRTK showed subcapsular fluid (*P* < 0.001). In the arterial phase, the proportion of slight enhancement in RCC was lower than that in the other two tumors (*P* = 0.007), and the proportion of marked enhancement was the highest (*P* = 0.002). In the venous phase, the proportion of slight enhancement in RCC was lower than that in the other two tumors (*P* < 0.001). Only CCSK had bone metastasis. There was no liver and lung metastasis in RCC.

**Conclusions:**

NWTs have their own imaging and clinical manifestations. CCSK can cause vertebral metastasis, MRTK can cause subcapsular effusion, and RCC tumor density is usually high and calcification. These diagnostic points can play a role in clinical diagnosis.

## Background

Malignant non-Wilms tumors (NWTs) in children include clear cell sarcoma of the kidney (CCSK), malignant rhabdomyoma tumor of the kidney (MRTK), renal cell carcinoma (RCC) and other extremely rare malignant tumors [1–3]. Preoperative identification of the tumor type relies primarily on imaging or diagnostic needle biopsy. Diagnostic puncture biopsy is an invasive examination with low repeatability and poor patient compliance. In daily diagnosis and treatment of tumor cases, imaging examination is an essential and noninvasive test that has a very crucial role in identifying the heterogeneity characteristics inside the tumor. With the continuous development of diagnostic imaging technology, the detection rate of renal tumors in children is high. However, when the tumor volume is large, it is difficult to distinguish renal tumors from primary adrenal tumors (such as neuroblastoma), but some studies have identified them by radionuclide imaging and achieved certain results [4, 5]. At present, there are difficulties in the identification of renal tumors. Although MR examination is safe and radiation free, it has not been well popularized within clinical practice. CT remains the imaging modality of choice, and low-dose scanning methods do not increase radiation doses to children. Because the pathology, treatment and prognosis of malignant NWTs are distinctly different [3, 6–10], it is necessary to conduct CT imaging examinations before surgery to determine differential diagnoses. The purpose of this study was to review the clinical and imaging features of malignant NWTs and features of tumor metastasis.

## Methods

### Patients

This study was a retrospective case–control investigation approved by the Ethics Committee of the hospital, and the requirement for the consent of patients was waived. The images of malignant NWTs confirmed through biopsy or surgical resection were searched from the picture archiving and communication system (PACS) of our hospital from March 2008 to July 2020. Inclusion criteria included: (1) Age younger than 18 years; (2) Primary unilateral renal tumor; (3) The pathological results were renal tumor; (4) The phase of the enhanced CT scan was intact; (5) Did not receive treatment before CT examination. The exclusion criteria were as follows: (1) pathological results obtained by tumor puncture only and (2) cases with failed CT image calling.

### Imaging examination

All patients underwent enhanced CT scans. In CT examinations, GE Healthcare 16 slice spiral CT, GE Healthcare 64 slice spiral CT and GE Discovery CT750 HD (General Electric Company, USA) were used. The scanning range was from the top of the diaphragm to the pelvic entrance. The thickness of the acquisition layer was 5 mm, the interval between layers was 5 mm, and the reconstruction interval was 0.625 mm. The patients were placed in the supine position, a nonenhanced CT scan was performed first to determine the lesion site, and then as enhanced scanning was performed. The contrast medium was iohexol (300 MGI/ml) at a dose of 1.1–1.6 ml/kg. The arterial phase was scanned 15–18 s after injection, and the venous phase was performed at 45–55 s.

Two pediatric imaging diagnostic doctors assessed the anatomical location, size, margin, homogeneity and contrast enhancement of the renal mass with respect to the normal renal parenchyma. CT images were reviewed and diagnosed independently by two radiologists and reread for cases with discordant diagnostic results.

### Statistics

Statistical analyses were performed using SPSS version 24.0 software (IBM Corp., Armonk, NY USA). Continuous and quantitative data (such as age and tumor diameter) were analyzed by one-way ANOVA and *P* values of less than 0.05 indicated that the difference was statistically significant. Categorical and qualitative data (such as gender, age group and image features) were analyzed by chi-square test of row × column data. When comparing the two groups of data, *P* values of less than 0.05 indicated that the difference was statistically significant. For pairwise comparisons among the three groups of data, *P* values of less than 0.017 indicate that the data difference was statistically significant.

## Results

Our study cohort included 65 patients with malignant NWTs. Twenty-two patients had CCSK, 27 patients had MRTK and 16 patients had RCC. In the current cohort, abdominal imaging was performed with enhanced CT in all patients. All enhanced CT scans included three phases, including the plain phase, arterial phase and venous phase. Each tumor was grouped according to age, named less than 1 year old, 1 to 4 years old, 5 to 9 years old and 10 to 14 years old. The demographic characteristics of the various tumors are shown in Table 1. The imaging characteristics of the NWT patient cohort at presentation are shown in Table 2.

**Table 1:** Demographic characteristics of the NWTs patient cohort at presentation

Parameter	CCSK	MRTK	RCC	*P* values	*P* values	*P* values	*P* values
	(n = 22)	(n = 27)	(n = 16)		C vs. M	C vs. R	M vs. R
Sex (M/F)	14/8 (1.75:1)	16/11 (1.45:1)	7/9 (0.78:1)	0.450	0.754	0.223	0.324
Age range (years)	0.6–10	0.3–6.2	2.9–14.6	< 0.001	0.012	< 0.001	< 0.001
Median age (years)	2.55	1.2	11.35				
*Age group (years)*							
< 1	4.5% (1/22)	44.4% (12/27)	0	< 0.001	0.002	0.291	< 0.001
1–4	77.4% (17/22)	51.9% (14/27)	12.5% (2/16)	< 0.001	0.066	< 0.001	0.010
5–9	13.6% (3/22)	3.7% (1/27)	25.0% (4/16)	0.118	0.460	0.375	0.107
10–14	4.5% (1/22)	0	62.5% (10/16)	< 0.001	0.202	< 0.001	< 0.001

*P* values indicates whether the difference between the three groups of tumors is statistically significant. C, CCSK; M, MRTK; R, RCC

**Table 2:** Imaging characteristics of the NWTs patient cohort at presentation

Parameter	CCSK	MRTK	RCC	*P* values	*P* values	*P* values	*P* values
	(n = 22)	(n = 27)	(n = 16)		C vs. M	C vs. R	M vs. R
Diameter (cm)	9.8 (range: 4.4 to 18.1)	5.9 (range: 1.5 to 11.1)	5.3 (range: 2.2 to 12.5)	< 0.001	< 0.001	< 0.001	0.462
Breaking through the renal fascia	5% (1/22)	26% (7/27)	19% (3/16)	0.136	0.044	0.158	0.590
Hemorrhage and necrosis	64% (14/22)	85% (23/27)	44% (7/16)	0.017	0.081	0.223	0.004
Calcification	14% (3/22)	7% (2/27)	44% (7/16)	0.009	0.809	0.037	0.005
Subcapsular fluid	0	33% (9/27)	0	< 0.001	< 0.001	/	0.002
*Arterial phase*							
Slight enhancement	68% (15/22)	70% (19/27)	25% (4/16)	0.007	0.869	0.007	0.004
Moderate enhancement	14% (3/22)	15% (4/27)	25% (4/16)	0.607	1.000	0.375	0.671
Marked enhancement	5% (1/22)	4% (1/27)	38% (6/16)	0.002	1.000	0.008	0.004
*Venous phase*							
Slight enhancement	77% (17/22)	56% (15/27)	6% (1/16)	< 0.001	0.112	< 0.001	0.001
Moderate enhancement	14% (3/22)	30% (8/27)	50% (8/16)	0.052	0.182	0.014	0.182
Clear enhancement	14% (3/22)	4% (1/27)	25% (4/16)	0.118	0.460	0.375	0.107
*Metastasis*							
Lymph node metastasis	9% (2/22)	19% (5/27)	19% (3/16)	0.603	0.348	0.388	0.985
Vein tumor thrombus	9% (2/22)	4% (1/27)	13% (2/16)	0.553	0.855	0.737	0.635
Bone (the vertebral body) metastasis	9% (2/22)	0	0	0.108	0.069	0.132	/
Liver and lung Metastases	5% (1/22)	22% (6/27)	0	0.022	0.079	0.291	0.013

*P* values indicates whether the difference between the three groups of tumors is statistically significant. C, CCSK; M, MRTK; R, RCC

### Demographic characteristics

There were no significant differences observed among the sex ratios of CCSK, MRTK and RCC (all *P* > 0.05) (Table 1), and the sex ratios (male vs. female) were 1.75:1, 1.45:1 and 0.78:1, respectively. For the onset age of tumors, there were significant differences observed among the groups (all *P* < 0.05) (Table 1). Among the three tumors, the onset age of MRTK patients was the smallest, while that of RCC patients was the largest. For the age group of onsets (Table 1), CCSK patients were distributed in all age groups. All MRTK patients were distributed in the age group under 10 years old, while RCC patients were mainly distributed in the age groups of 5–9 and 10–14. For the less than 1 age group, the proportion of patients with MRTK was larger than that with CCSK and RCC (*P* = 0.002 and *P* < 0.001, respectively), while the proportion of patients with CCSK and RCC was similar (*P* = 0.291). For the 1–4 age group, the proportion of RCC patients was lower than that of the other two groups (*P* < 0.001 and *P* = 0.010, respectively), while the proportion of patients with CCSK and MRTK was similar (*P* = 0.066). For the 5–9 age group, there were no significant differences observed among the groups (all *P* > 0.05). For the 10–14 age group, the proportion of RCC patients was larger than that of the other two groups (all *P* < 0.001), while the proportion of patients with CCSK and MRTK was similar (*P* = 0.202).

### Imaging findings

#### Primary mass

**Fig. 1:**
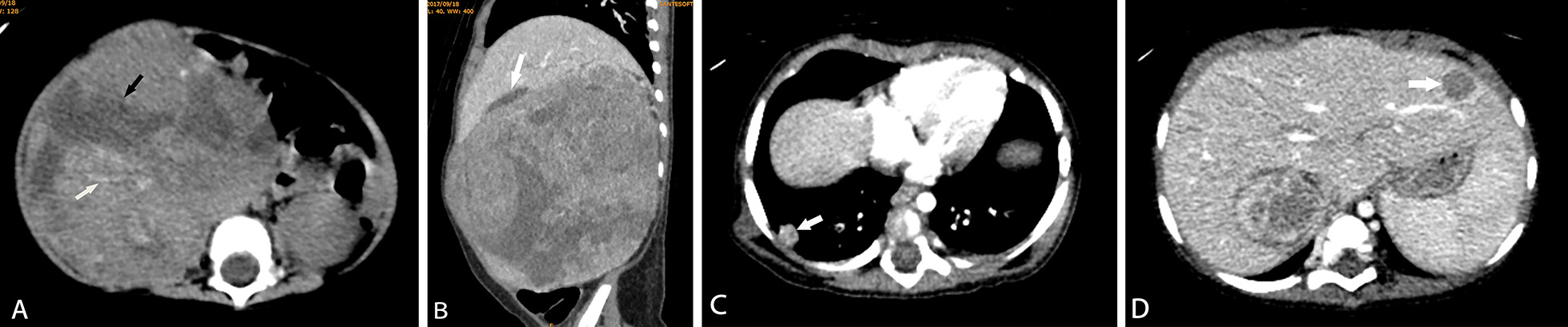
MRTK in a 7-month-old girl. **A** Large necrotic areas (black arrow) and hemorrhagic lesions (white arrow) were visible within the tumor, and the tumor density was uneven. **B** The sagittal reconstructed image showed subcapsular effusion (white arrow) at the edge of the tumor. **C**, **D** Tumor metastasis (white arrow) was found in the lung and liver

**Fig. 2:**
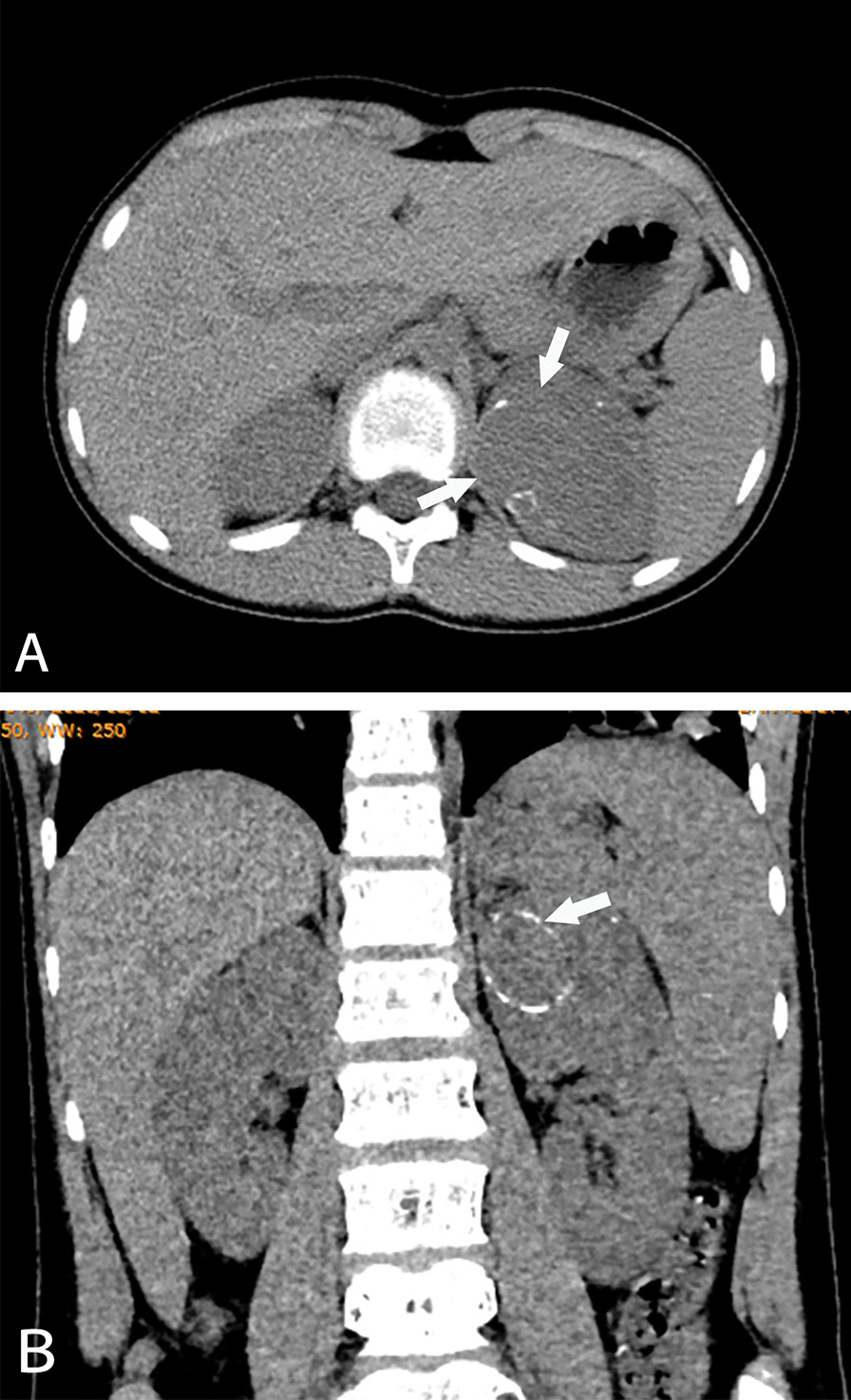
RCC in an 11-year-old boy. **A** The internal density of the left renal tumor was uniform, and the tumor density did not decrease (white arrow). **B** Rim calcification (white arrow) was seen in the tumor

The tumor diameter of the CCSK was larger than that of MRTK and RCC (all *P* < 0.001), while the tumor diameter of the MRTK and RCC was not significantly different (*P* = 0.462) (Table 2). CCSK, MRTK and RCC could break through the renal fascia, but no significant differences were observed (*P* = 0.136). However, MRTK was more likely to break through the renal fascia than the other two tumors. For hemorrhage and necrosis, the proportion of MRTK patients was larger than that of the other two tumors (*P* = 0.017) (Fig. 1a). The proportion of hemorrhage and necrosis in RCC was lowest, so the overall density of RCC was higher than that of CCSK and MRTK (Fig. 2a). For calcification in tumors, the proportion of calcification in RCC was highest (*P* = 0.009) (Fig. 2b), while there was no significant difference in the incidence of tumor calcification between CCSK and MRTK (*P* = 0.809). Among the three tumors, only MRTK tumors showed subcapsular fluid (Fig. 1b), accounting for approximately 33% (*P* < 0.001).

**Fig. 3:**
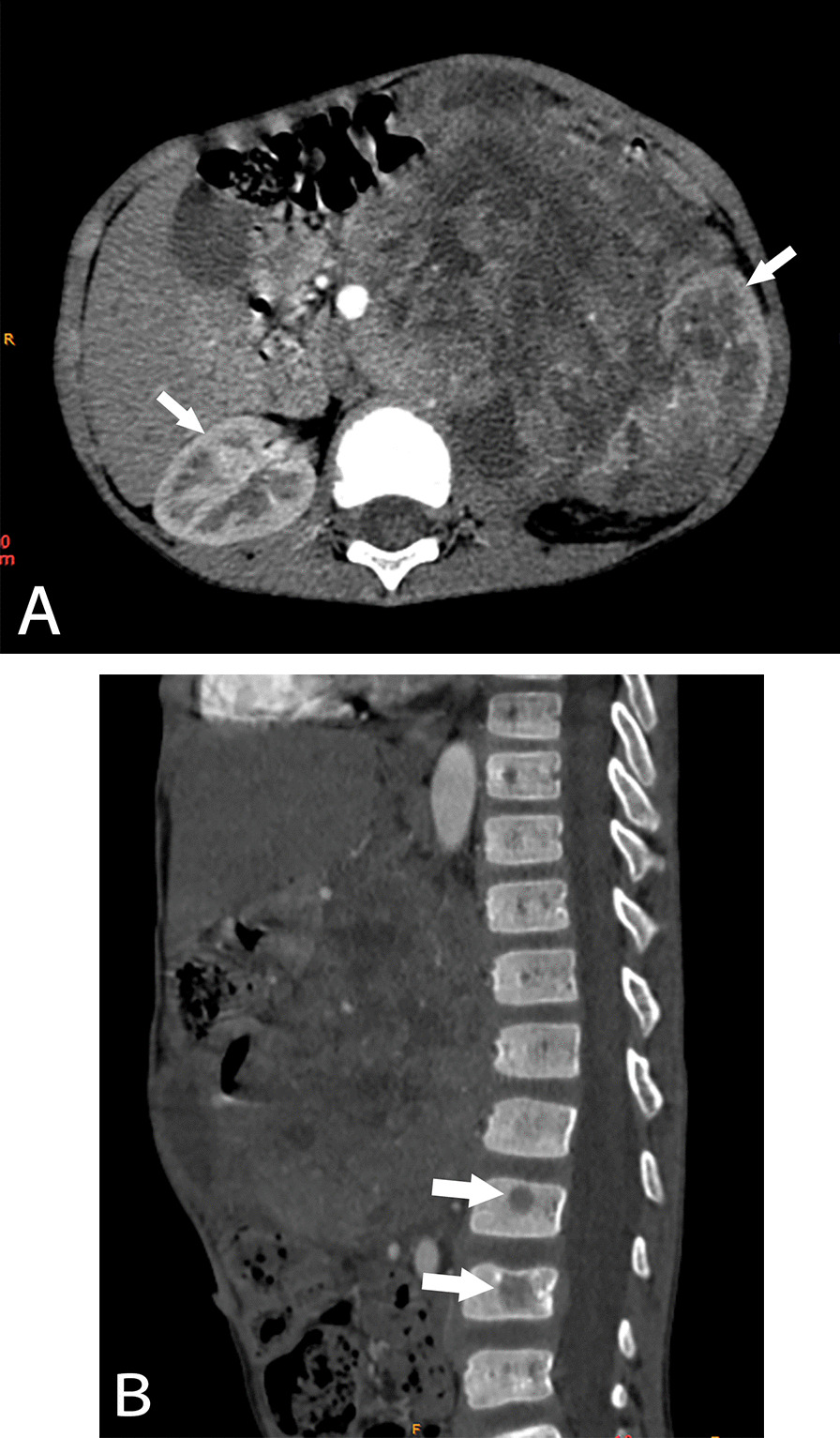
CCSK in a 5-year-old boy. **A** Large tumor tissue was seen in the left kidney area, and the density of the tumor was uneven. Tumor tissue showed slight enhancement, less than the normal renal parenchyma (white arrow). **B** Sagittal bone window images showed low-density lesions (white arrow) in lumbar vertebrae, suggesting tumor metastasis

The enhancement characteristics of CCSK, MRTK and RCC tumors were different. In the arterial phase, only 25% of RCCs showed slight enhancement, which was lower than that of CCSK and MRTK (*P* = 0.007 and *P* = 0.004, respectively), while there was no significant difference in the proportion of mild enhancement between CCSK and MRTK (*P* = 0.869) (Fig. 3a). However, the proportion of marked enhancement in RCC was the highest among the three tumors, accounting for approximately 38% (*P* = 0.002; CCSK vs. MRTK, *P* = 0.008; MRTK vs. RCC, *P* = 0.004). There were no significant differences observed among the patients with moderate enhancement (all *P* > 0.05). In the venous phase, the proportion of slight enhancement in RCC was lowest, only 6% (*P* < 0.001), while there was no significant difference in the proportion of slight enhancement between CCSK and MRTK (*P* = 0.112). The proportion of moderate enhancement in RCC was higher (50%; CCSK vs. RCC, *P* = 0.014). There were no significant differences observed among the patients with clear enhancement (all *P* > 0.05).

#### Metastases based on imaging

The metastasis characteristics of the three tumors were also different. For lymph node metastasis and vein tumor thrombus, there were no significant differences observed among the three types of tumors (all *P* > 0.05). Among the three types of tumors, only CCSK had bone metastasis (9%), and the tumor metastasized to vertebral bone (Fig. 3b). Both CCSK and MRTK had liver and lung metastasis, but the proportion of MRTK was higher, accounting for 22% (Fig. 1c and 1d).

## Discussion

Renal tumors in children mainly originate in the posterior renal germ or posterior interrenal lobe, which are common abdominal tumors in children. Malignant renal tumors accounted for 5.2% of malignant tumors in children [11]. NWTs account for approximately 10% of pediatric renal tumors. However, there are many kinds of tumors, such as CCSK, MRTK, RCC and other extremely rare malignant tumors [1, 12–16].

CCSK is a rare malignant renal tumor that primarily occurs in children and was first described by Kidd et al. in 1970 [17]. CCSK has recently been regarded as a malignancy distinct from Wilms tumors (WTs) [12, 15]. CCSK represents approximately 4% of all childhood renal neoplasms and has a marked male predilection. CCSK is commonly diagnosed in children 1 to 4 years of age [2]. In our CCSK study, the ratio of males to females was approximately 1.75:1, and the median age of onset was 3.1 years (range of 0.6 to 10 years). The results of our study were consistent with the literature. MRTK is an aggressive embryonal tumor that arises from primitive cells in the renal medulla and frequently involves the hilum and collecting system. MRTK accounts for less than 2% of childhood renal tumors [13]. MRTK was originally described in the kidneys of young children by Beckwith and Palmer in 1978 and is considered to be the most aggressive malignant renal tumor in childhood [18]. The median age of patients with renal RT ranges from 11 to 18 months, with a mean age of 11 to 18 months [13, 19, 20]. MRTK affects boys slightly more commonly than girls (1.5:1) [13, 20]. In our study, the ratio of males to females was approximately 1.45:1, and the median age of onset was approximately 1.4 years (range of 0.3 to 6.2 years). Therefore, if the child is younger (usually less than 1 year old), MRTK should be considered first [1]. Although well recognized, RCC is an uncommon tumor, as it is the second most common renal malignancy diagnosed among pediatric and adolescent patients, accounting for 2% to 6% of renal cancers [1, 7, 14]. The onset age of RCC is usually older, generally more than 10 years old, with an average age of 11–12 years [11, 14, 21]. In our study, the median age of RCC onset was 10.1 years, ranging from 2.9 to 14.6 years, but most patients were over 10 years old. The age of RCC onset is significantly different from that of CCSK and MRTK, which is also one of the important differentiation points of the three renal tumor types.

CCSK is usually highly malignant, so it is of great significance to identify it from NWTs [1, 12]. CCSK is usually unilateral, with necrosis and cystic lesions of different sizes and numbers. Hemorrhage and necrosis are frequent findings [22]. The blood supply of the tumor is abundant. Enhanced scans can have mild to moderate enhancement (> 20 Hu), and approximately 25% have calcification [1, 23]. In our study, CCSK tumors did not easily break through the renal fascia. More than half of the tumors showed evidence of hemorrhage and necrosis but rarely showed calcification. Most CCSK tumors had mild enhancement, which may be related to CCSK not easily invading blood vessels [13]. The results of our study are similar to those in the literature. Although CCSK is a highly malignant tumor, only one CCSK tumor broke through the renal fascia in our study, which may be one of the differentiation points between CCSK and other tumors. At the same time, our study also found that the tumor volume of CCSK is generally larger than that of MRTK and RCC, which can distinguish CCSK.

The CT findings of MRTK are relatively nonspecific, but some imaging features can also prompt the diagnosis. The MRTK tumors are relatively large and heterogeneous. Tumors in the central area often involve the renal hilum. Most of these tumors are accompanied by hemorrhage, necrosis and subcapsular effusion [1, 13]. Subcapsular effusion is a more specific imaging manifestation, which further confirms the characteristics of the MRTK tumor hemorrhage and necrosis [1, 13]. In our study, 85% of the tumors had hemorrhage and necrosis, and approximately 33% of the tumors had subcapsular effusion. These two factors can be used as important factors in the diagnosis of MRTK. An enhanced CT scan showed uneven enhancement of the tumor, and the enhancement degree was lower than that of muscle tissue [13]. Approximately 85% of the tumors in our study showed mild to moderate enhancement. Approximately 10% to 20% of affected children have primary brain tumors [13, 18, 24]. However, some studies have suggested that brain tumors are metastatic tumors rather than primary tumors [13, 25]. In our study, only 2 patients had tumor lesions in the brain, so the diagnosis of brain tumors as metastatic tumors remains to be studied.

In CT imaging, the density of RCC was higher than that of the normal renal parenchyma, which was separate from CCSK and MRTK. Calcification, hemorrhage and necrosis can be found in most RCC tumors [1, 14, 26, 27]. In our study, approximately 44% of the tumors showed evidence of hemorrhage and necrosis, and approximately 44% of the tumors showed calcification. However, calcification was rare in CCSK and MRTK. In contrast-enhanced CT imaging, approximately 63% of the tumors showed moderate to clear enhancement, which is also different from the enhancement degree of CCSK and MRTK. This finding is not consistent with the known literature results [14], which may be related to the sample size of patients because approximately 25% of the tumors in our study showed slight enhancement. At present, some studies have used MRI images combined with radiomics to predict the Fuhrman classification of RCC in adult patients and have achieved good results [28]. This study suggests that we can use radiomics or deep learning methods to predict the type of renal tumors in the future and believe that it will achieve better results combined with image features.

CCSK, MRTK and RCC have different characteristics in tumor metastasis. CCSK is well known for its aggressive nature. Bone metastasis is a prominent feature of CCSK [16, 22, 29]. However, data from the American National Wilms Tumor Study suggest that bone metastases are infrequent in CCSK, and indeed, bone metastases accounted for only 6% of metastases among 351 patients [6]. In our study, approximately 9% of tumors developed bone metastasis (the vertebral body). Bone metastasis is a prominent feature of CCSK. If bone metastasis occurs (especially vertebral metastasis), the first consideration should be CCSK rather than other renal tumors. Although CCSK tumors do not easily break through the renal fascia, they still metastasize locoregionally and distantly, which further indicates their invasiveness. Lung metastasis is most common in MRTK, which is also different from other renal tumors, including WTs [22, 30]. In our study, approximately 22% of tumors developed pulmonary metastases, indicating that MRTK tumors mainly metastasize distantly. It is true that lung metastases in very young patients could be an indication of MRTK, where a biopsy should be considered. In the literature, distant metastasis of RCC tumors is not characteristic [31]. In our study, no distant organ metastasis was found in RCC tumors, indicating that RCC tumors mainly metastasize locoregionally. Lymph node metastasis is not specific in the differential diagnosis of renal tumors.

This study had several limitations. Most of the cases in this study belonged to the previous cases, when MR examination was not yet in clinical popularity, so most of the patients underwent CT examination. With the popularity of MR in recent years, MR has been gradually applied in the clinic to evaluate renal tumors. However, we believe that CT-related studies will be instructive for the future study of MR, and at the same time, CT findings will have important clinical implications for the evaluation of tumor metastasis.

## Conclusions

CCSK, MRTK and RCC have their own imaging and clinical manifestations. The volume of CCSK tumors is large, most of the enhanced scans show slight enhancement, and vertebral metastases can occur. The onset age of MRTK was younger, and hemorrhage and necrosis were common in the tumor. Subcapsular effusion was seen in most cases of MRTK. Most MRTK patients showed slight to moderate enhancement in enhanced scanning, and distant metastases, such as liver and lung metastases, were common. The onset age of RCC is older. Hemorrhage and necrosis within the tumor are relatively rare, and calcification is more common. Enhanced scans are mostly moderate and clear enhancement. The different clinical and imaging features of CCSK, MRTK and RCC can provide help for the diagnosis and treatment of tumors.

## Data Availability

The datasets used and analysed in the current study are available from the corresponding author on reasonable request.

## Abbreviations

WTs: Wilms tumors
NWTs: Non-Wilms tumors
CCSK: Clear cell sarcoma of the kidney
MRTK: Malignant rhabdomyoma tumor of the kidney
RCC: Renal cell carcinoma
PACS: Picture archiving and communication system
